# AMPLIFYING THE POSITIVE: RECIPROCAL ASSOCIATIONS BETWEEN SAVORING AND POSITIVE AFFECT

**DOI:** 10.1093/geroni/igae098.1482

**Published:** 2024-12-31

**Authors:** Jacquelyn Stephens, Jennifer Smith

**Affiliations:** Mather Institute, Evanston, Illinois, United States; Mather Institute, Evanston, Illinois, United States

## Abstract

Savoring and positive affect are closely related, yet little work has disentangled the bi-directional relationship between them, as they have primarily been examined cross-sectionally. Further, more empirical work is needed to show that savoring is longitudinally associated with increased positive affect even when accounting for baseline positive affect. We examined year-to-year associations between savoring and positive affect using three annual waves of data from the Age Well study. Participants consisted of 2945 older adults (Mage = 82.08, SD = 6.38; 66% female) recruited from Life Plan Communities across the United States. Positive affect was modeled on savoring ability using random-intercept cross-lagged panel models. Our results revealed significant bi-directional relationships, such that savoring predicted later positive affect (B =.15, p >.001) and positive affect predicted later savoring (B =.34, p >.001). Our results show evidence that changes in savoring sequentially predict greater positive affect, as well as the reverse. In line with views of upward spirals of positive emotion, increasing positive emotion regulation indeed contributes to greater emotional well-being in older adults, independent of initial levels of positive emotion.

